# Using Bidimensional Multiscale Entropy Analysis of Ultrasound Images to Assess the Effect of Various Walking Intensities on Plantar Soft Tissues

**DOI:** 10.3390/e23030264

**Published:** 2021-02-24

**Authors:** Ben-Yi Liau, Fu-Lien Wu, Keying Zhang, Chi-Wen Lung, Chunmei Cao, Yih-Kuen Jan

**Affiliations:** 1 Department of Biomedical Engineering, Hungkuang University, Taichung 433304, Taiwan; byliau@hk.edu.tw; 2 Rehabilitation Engineering Lab, Department of Kinesiology and Community Health, University of Illinois at Urbana-Champaign, Champaign, IL 61820, USA; fulienwu@illinois.edu (F.-L.W.); keyingz@illinois.edu (K.Z.); lung@illinois.edu (C.-W.L.); 3 Department of Creative Product Design, Asia University, Taichung 41354, Taiwan; 4 Division of Sports Science and Physical Education, Tsinghua University, Beijing 100084, China; caocm@tsinghua.edu.cn

**Keywords:** complexity, multiscale entropy, plantar foot, regularity, ultrasound

## Abstract

Walking performance is usually assessed by linear analysis of walking outcome measures. However, human movements consist of both linear and nonlinear complexity components. The purpose of this study was to use bidimensional multiscale entropy analysis of ultrasound images to evaluate the effects of various walking intensities on plantar soft tissues. Twelve participants were recruited to perform six walking protocols, consisting of three speeds (slow at 1.8 mph, moderate at 3.6 mph, and fast at 5.4 mph) for two durations (10 and 20 min). A B-mode ultrasound was used to assess plantar soft tissues before and after six walking protocols. Bidimensional multiscale entropy (MSE_2D_) and the Complexity Index (CI) were used to quantify the changes in irregularity of the ultrasound images of the plantar soft tissues. The results showed that the CI of ultrasound images after 20 min walking increased when compared to before walking (CI_4_: 0.39 vs. 0.35; CI_5_: 0.48 vs. 0.43, *p* < 0.05). When comparing 20 and 10 min walking protocols at 3.6 mph, the CI was higher after 20 min walking than after 10 min walking (CI_4_: 0.39 vs. 0.36, *p* < 0.05; and CI_5_: 0.48 vs. 0.44, *p* < 0.05). This is the first study to use bidimensional multiscale entropy analysis of ultrasound images to assess plantar soft tissues after various walking intensities.

## 1. Introduction

Walking as a physical activity intervention has been universally recommended for improving physical and psychological health [1]. In terms of walking volume (i.e., intensity and duration), the physical activity guidelines for Americans suggest that people should perform moderate-intensity physical activity over 150 min or vigorous-intensity physical activity over 75 min per week [1]. The minimum duration of a bout of aerobic physical activity (e.g., walking) is 10 min [1]. However, these physical activities cause prolonged high plantar pressures and shear stresses that may cause foot ulcers [2,3,4]. In order to prescribe a safe exercise regimen for improving health and reducing risk for foot ulcers, it is essential to investigate the effects of different walking speeds and durations on plantar soft tissues [5,6,7].

Soft tissue thickness of the plantar foot has been widely investigated using ultrasound [8,9,10,11,12]. Research studies demonstrated that people with diabetes mellitus have thinner plantar soft tissue, especially under the first and second metatarsal head regions. The altered thickness of plantar soft tissue has been identified as a causative factor of foot ulcers [10,11,12]. Decreased soft tissue thickness may increase the peak plantar pressure, subsequently contributing to foot ulcers. However, this soft tissue biomechanical property cannot be fully characterized by the thickness. Moreover, measuring the thickness of plantar soft tissue using ultrasound relies on the experience of the operator [13]. Thus, an objective analysis of ultrasound images is necessary for clinical assessment and diagnosis.

Entropy methods to distinguish pathological soft tissues from normal ones have been employed in various conditions, including muscles with myofascial trigger points [14], muscle fatigue [15], myocardial contusions [16,17], and malignant hepatic lesions [18]. Ultrasound images taken from these different soft tissue conditions demonstrate different image textures that may not be detected by clinicians’ visual inspections [13]. Thus, the entropy method, a measure of heterogeneity, could be implemented to quantify these changes shown in ultrasound images. In our case, applying an entropy-based method to analyze B-mode ultrasound images of plantar soft tissues may be used to evaluate the effect of various intensities of physical activity on plantar soft tissues.

Costa et al. proposed the algorithm of multiscale entropy (MSE) to estimate the complexity degree of signals using a range of temporal scales [19,20]. The wide applications of MSE in medical diagnosis and analysis are due to its ability to correct erratic estimations of some pathological conditions using traditional, entropy-based methods. MSE can characterize the complexity of physiological time-series signals using multiple scales. Recently, bidimensional multiscale entropy (MSE_2D_) was introduced by Silva et al. to overcome the limitations of MSE when quantifying the complexity of images [21,22,23]. The MSE_2D_ algorithm is an extension of MSE to bidimensional (two-dimensional) data, such as biomedical images [21,22,23]. This new method has not been used in various pathophysiological conditions. To the best of our knowledge, the MSE_2D_ method has not been applied when assessing ultrasound images of plantar soft tissues.

The objective of this study was to apply the newly developed method of bidimensional multiscale entropy to assess complexity changes of plantar ultrasound images after walking at different speeds and durations. To the best of our knowledge, this is the first study to use bidimensional multiscale entropy to investigate gait performance. The findings from bidimensional multiscale entropy could be used to better understand the effect of various walking intensities on plantar soft tissue as assessed by ultrasound.

## 2. Materials and Methods

A 3 × 2 factorial design including three speeds (slow at 1.8 mph, moderate at 3.6 mph, and fast at 5.4 mph) and two durations (10 and 20 min) was used in this study. This study was part of a larger research study investigating the biomechanical response of the plantar foot to various walking intensities [5]. The three speeds were selected to simulate three common speeds in adults, including slow walking at 1.8 mph, preferred fast walking speeds at 3.6 mph, and slow running at 5.4 mph. The selection of 10 min and 20 min durations was based on the common duration of walking and physical activities [5].

### 2.1. Subjects

The inclusion criterion was age between 18 and 35 years. The exclusion criteria were active foot ulcers, diabetes, vascular diseases, hypertension, inability to walk for 20 min independently, inability to walk at the speed of 5.4 mph independently, or the use of vasoactive medications. Each subject signed the informed consent approved by the University of Illinois at Urbana-Champaign Institutional Review Board before the experimental procedures. All procedures were performed in the Rehabilitation Engineering Laboratory at the University of Illinois at Urbana-Champaign. Room temperature was maintained at 24 ± 2 °C. The participants relaxed in a supine position for at least 30 min prior to testing.

### 2.2. Instruments

**Figure 1:**
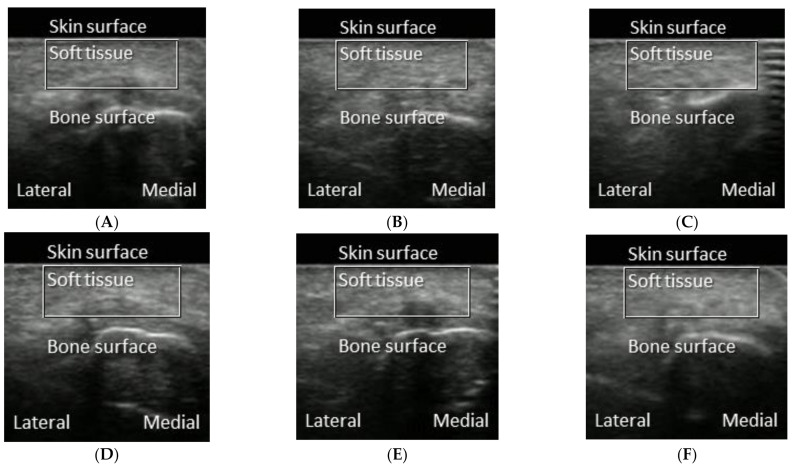
Examples of B-mode ultrasound images obtained from the first metatarsal head of a participant after different walking conditions. (**A**) 10 min duration at 1.8 mph. (**B**) 10 min duration at 3.6 mph. (**C**) 10 min duration at 5.4 mph. (**D**) 20 min duration at 1.8 mph. (**E**) 20 min duration at 3.6 mph. (**F**) 20 min duration at 5.4 mph. Each image shows the region of interest (ROI, from the surface of the skin to the surface of the first metatarsal head with a width of 100 pixels) and the medial and lateral sides of the participant.

Plantar soft tissue images were measured by an ultrasound device (Aloka Pro Sound Alpha 7, Hitachi-Aloka Medical, Ltd., Tokyo, Japan) with a linear array transducer (UST-5412; frequency range: 5–13 MHz; axial resolution: 0.25 mm, Aloka, Tokyo, Japan) [24]. The B-mode ultrasound images of the right first metatarsal head of the participants were obtained by the same operator. The B-mode ultrasound refers to the two-dimensional ultrasound image representing ultrasound echoes. The brightness of each pixel is determined by the amplitude of the returned echo signal. The selection of a B-mode ultrasound device was due to its low cost compared to high-end ultrasound devices (e.g., shear wave elastography). However, an interpretation of B-mode ultrasound images requires extensive training. In this study, we explored the use of MSE_2D_ to quantify the changes of plantar ultrasound images after walking at various intensities. The region of interest was defined as a rectangular area from the skin surface to the surface of the first metatarsal head with a width of 100 pixels. Examples of B-mode ultrasound images of the soft tissue at the first metatarsal head after six walking intensities are provided in Figure 1. These B-mode images do not exhibit clear differences. The ultrasound images of plantar soft tissues were saved in the Digital Imaging and Communications in Medicine (DICOM) format for further analysis.

### 2.3. Experimental Procedures

The plantar soft tissue was measured using the ultrasound device before and immediately after each of six walking protocols when the participant was in a supine position. The subjects wore their shoes and walked on the treadmill at a speed of 1.8 mph for either 10 or 20 min at the first visit. A crossover design was used to randomly assign subjects to either a 10 min or 20 min walking protocol. The washout period was 20 min to minimize the carryover effects between two durations of walking. All subjects returned to the lab to perform 3.6 mph and 5.4 mph walking protocols at the second and third visits, respectively. Each visit was separated between 7 ± 2 days.

### 2.4. Bidimensional Multiscale Entropy Analysis

The multiscale entropy (MSE) uses the algorithm of sample entropy to estimate the regularity of different time scales based on the approximate entropy [19,20,25,26]. MSE_2D_ [21,22,23] is an extension of the one-dimensional MSE and is composed of two steps:

(i) Coarse granulation is carried out for the given embedded dimension *m* and similarity tolerance *r.* An image **u**(**i**, **j**) be considered with width W and height H; **x_m_**(**i**, **j**) is a set of pixels of *u* ranging columns *j* to *j* + *m* − 1 and lines *i* to *i* + *m* − 1. The 2D coarse-graining procedure $y_{i,j}^{\left(\tau \right)}$ can be derived by Equation (1).
  (1) $$y_{i,j}^{\left(\tau \right)}=\frac{1}{\tau^{2}}\overset{k=i\tau }{\underset{k=\left(i-1\right)\tau +1}{\sum }}\overset{l=j\tau }{\underset{l=\left(j-1\right)\tau +1}{\sum }}u_{k,l}$$

where $1\leq i\leq \frac{H}{\tau }$ and $1\leq j\leq \frac{W}{\tau }$. *τ* is the scale factor. For *τ = 1*, the coarse-grained image {y^(1)^} corresponds to the original image. The width of the coarse-grained image $\left\{y^{\left(\tau \right)}\right\}$ is $\frac{W}{\tau }$ and the height is $\frac{H}{\tau }$.

For an image u, let **x_m_**(**i**, **j**) be the m-length square window with origin at **u**(**i**, **j**): **x_m_** (**i**, **j**) is the set of pixels *u* ranging columns *j* to *j* + *m* − 1 and lines *i* to *i* + *m* − 1, which **x_m_**(**i**, **j**) = [**u**(**i**, **j**), **u**(**i**, **j** + 1),…, **u**(**i**, **j** + **m** − 1), **u**(**i** + 1, **j**), **u**(**i** + 1, **j** + 1),…, **u**(**i** + 1, **j** + **m** − 1),…, **u**(**i** + **m** − 1, **j** + **m** − 1)]. Let **N_m_** be the total number of square windows within *u* that can be generated for both *m* and *m* + 1 size: **N_m_** = (**W** − **m**) × (**H** − **m**).

(i) By the coarse-grained procedure, two-dimensional sample entropy (SampEn_2D_) for a similarity threshold r could be estimated by
  (2) $${\mathrm{SampEn}}_{2D}=-\ln\frac{U^{m+1}\left(r\right)}{U^{m}\left(r\right)}$$

where

$$U^{m}\left(r\right)=\frac{1}{N_{m}}\overset{i=H-m;j=W-m}{\underset{i=1;j=1}{\sum }}U_{i,j}^{m}\left(r\right)\;U^{m+1}\left(r\right)=\frac{1}{N_{m}}\overset{i=H-m;j=W-m}{\underset{i=1;j=1}{\sum }}U_{i,j}^{m+1}\left(r\right)$$

and

$$U_{i,j}^{m}\left(r\right)=\frac{\left[\#{\;\mathrm{of}\;x}_{m}\left(a,b\right)\left|d\left[x_{m}\left(i,\;j\right),{\;x}_{m\;}\left(a,b\right)\right]\leq r\right.\right]}{N_{m}-1}\;U_{i,j}^{m+1}\left(r\right)=\frac{\left[\#{\;\mathrm{of}\;x}_{m+1}\left(a,b\right)\left|d\left[x_{m+1}\left(i,\;j\right),{\;x}_{m+1\;}\left(a,b\right)\right]\leq r\right.\right]}{N_{m}-1}$$

where a and b range from 1 to *H-m* and from 1 to *W-m*, respectively, and (a, b) ≠ (i, j), distance d is defined as $d\left[x_{m\;}\left(i,j\right),\;x_{m}\left(a,b\right)\right]=max\left(\left|u\left(i+k,j+l\right)-u\left(a+k,b+l\right)\right|\right)$, where *k* and *l* range from 0 to *m − 1*. According to the report in a previous study, in general, *m* = 2 is suggested [19]. On the other hand, *r* is a similarity threshold for MSE_2D_ estimation. However, *r* was not easy to determine due to its specification. We have tested several values of *r,* and it was found that *r* = 0.05 was more sensitive in showing obvious differences. Therefore, *m* = 2 and *r* = 0.05 were the settings in this study.

Therefore, MSE_2D_ with the scale factor τ can be defined by Equation (3)

(3) $${\mathrm{MSE}}_{2D}\left(u,\tau ,m,r\right)={\mathrm{SampEn}}_{2D}\left(y^{\left(\tau \right)},m,r\right)$$

The complexity index (CI) is the summation of SampEn_2D_ from the scale factor 1 to the maximum:

(4) $${\mathrm{CI}}_{\tau }=\overset{\tau }{\underset{i=1}{\sum }}{\mathrm{SampEn}}_{2D}\left(i\right)$$

The baseline demographic data were reported with descriptive statistics. The 3 × 2 two-way analysis of variance (ANOVA) with repeated measures was used to compare the CI between the three speeds (1.8, 3.6, and 5.4 mph) and two durations (10 and 20 min) and the interaction between speeds and durations. A one-way ANOVA with Fisher’s least significant difference correction was used for pairwise comparisons of the CI between three walking speeds (1.8, 3.6, and 5.4 mph) under each walking duration (10 and 20 min). The correction was used to overcome multiple comparison issues in this repeated measures study. The significance level was set to 0.05. All statistical tests were performed using SPSS 26 (IBM, Somers, NY, USA). The CI was calculated by the MATLAB R2019b (MathWorks, Inc., Natick, MA, USA).

## 3. Results

Twelve healthy subjects (five males, seven females) were recruited in this study. The demographic data were (mean ± standard deviation): age, 27.1 ± 5.8 years; height, 170.3 ± 10.0 cm; and weight, 63.5 ± 13.5 kg.

### 3.1. Walking Speed Effect on MSE_2D_ Values

**Figure 2:**
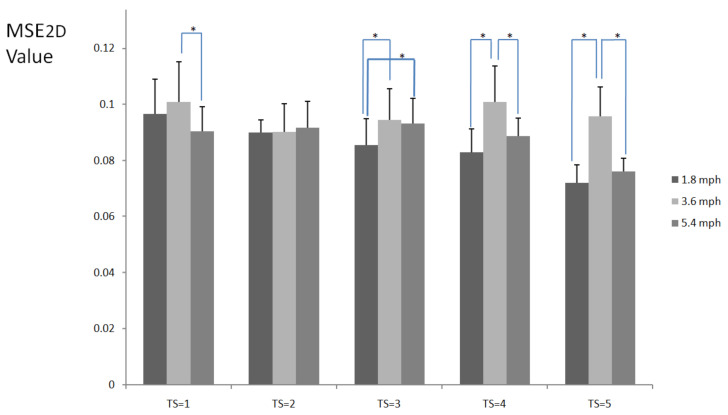
MSE_2D_ values of plantar ultrasound images after walking at 1.8, 3.6, and 5.4 mph for 20 min at the time scales 1, 2, 3, 4, and 5. Data are presented as mean + standard deviation. The symbol * indicates *p* < 0.05.

**Figure 3:**
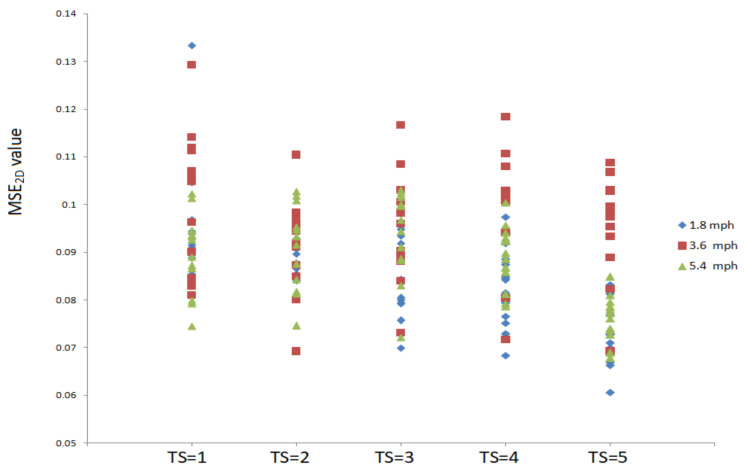
All participants’ MSE_2D_ values of plantar ultrasound images after walking at 1.8, 3.6, and 5.4 mph for 20 min at the time scales 1, 2, 3, 4, and 5.

Figure 2 shows MSE_2D_ values of plantar ultrasound images after three walking speeds for 20 min. MSE_2D_ values significantly increased after walking at 3.6 mph at the time scales 1, 3, 4, and 5 compared to walking at 1.8 mph (*p* < 0.05). MSE_2D_ values at 3.6 mph were higher than those at 5.4 mph at the time scales 4 and 5 (*p* < 0.05). All participants’ data are presented in Figure 3.

### 3.2. Walking Duration Effect on MSE_2D_ Values

**Figure 4:**
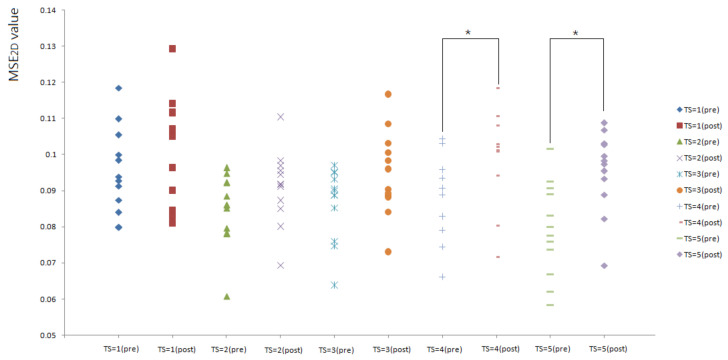
All participants’ MSE_2D_ values of plantar ultrasound images before and after walking at 3.6 mph for 20 min. The symbol * indicates *p* < 0.05.

Figure 4 shows the comparison of MSE_2D_ values of plantar ultrasound images before and after walking at 3.6 mph. In this study, only MSE_2D_ values of plantar ultrasound image before and after walking at 3.6 mph for 20 min showed significant differences at the time scales 4 and 5 (*p* < 0.05). After walking at 3.6 mph for 20 min, plantar soft tissue significantly exhibited higher complexity.

### 3.3. Complexity Index after 20 min Walking

**Figure 5:**
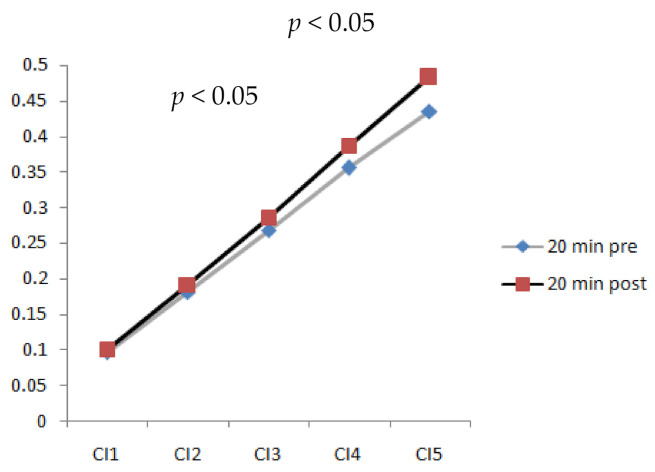
Comparison of complexity index values at time scales 1, 2, 3, 4, and 5 of plantar ultrasound images before and after walking at 3.6 mph for 20 min (CI_4_, CI_5_, *p* < 0.05).

The results indicated that a longer walking duration (20 min) significantly increased the complexity index (CI) of plantar soft tissues (*p* < 0.05). Figure 5 shows CI values at different time scales after walking at 3.6 mph for 20 min. It shows a trend wherein the complexity index increased as the time scales increased. The values of CI_4_ (complexity index at time scale 4) and CI_5_ (complexity index at time scale 5) indicated a significant difference (*p* < 0.05). Time scales 4 and 5 were able to detect the differences in plantar ultrasound images before and after walking.

### 3.4. Walking Duration and Speed Effect on Complexity Index

**Figure 6:**
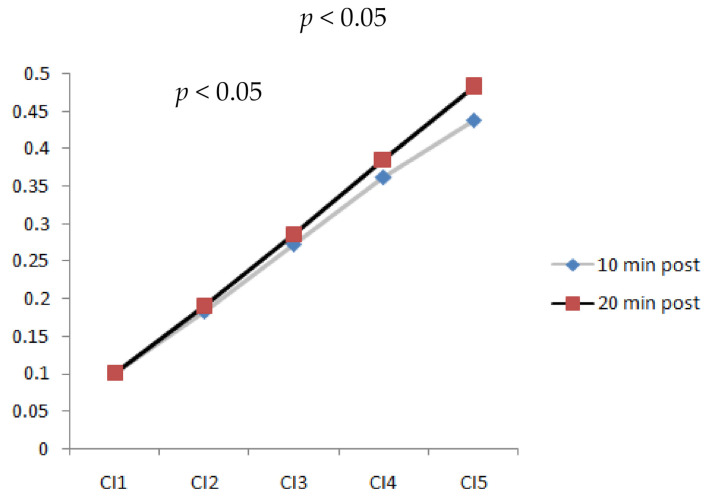
Comparison of complexity index values at time scales 1, 2, 3, 4, and 5 of plantar ultrasound images after 10 min and 20 min walking at 3.6 mph (CI_4_, CI_5_, *p* < 0.05).

Figure 6 shows the trend of CI change from time scales 1 to 5 for both walking durations of 20 and 10 min. At time scales 1, 2, and 3, no differences existed for the walking durations. However, differences for the walking durations significantly increased (*p* < 0.05) in CI_4_ and CI_5_ while walking at a speed of 3.6 mph. According to the results, longer walking durations led to higher irregularity in the images from the metatarsal soft tissue. However, walking slower and faster did not significantly change the complexity of different walking durations. Therefore, a walking speed of 3.6 mph could be critical due to its effects on properties of the soft tissue.

**Figure 7:**
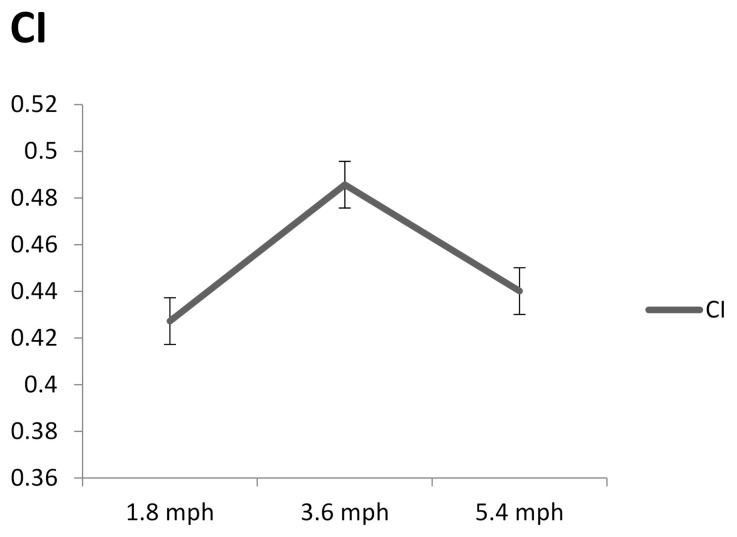
The complexity index after walking at 1.8, 3.6, and 5.4 mph for 20 min. Data are presented as mean + standard deviation.

Figure 7 shows the complexity index of plantar ultrasound images after walking at 1.8, 3.6, and 5.4 mph for 20 min. The complexity index of walking at 3.6 mph was significantly higher than 1.8 (*p* < 0.05) and 5.4 mph (*p* < 0.05).

## 4. Discussion

Bidimensional multiscale entropy was used to assess the complexity of ultrasound images of the plantar foot in response to different walking speeds (slow at 1.8 mph, moderate at 3.6 mph, and fast at 5.4 mph) and durations (10 and 20 min). Our results demonstrated that MSE_2D_ and complexity index values increased significantly after 20 min walking compared to 10 min walking, and the MSE_2D_ value at 3.6 mph was significantly higher than at 1.8 mph and 5.4 mph after 20 min walking. Among these six walking conditions, walking at 3.6 mph for 20 min resulted in the highest complexity of plantar ultrasound images. This is the first study to apply bidimensional multiscale entropy to quantify plantar ultrasound images.

One of the study’s purposes was to investigate how walking speed and duration influence the plantar soft tissue from the perspective of ultrasound image complexity. The current results show that walking at 3.6 mph for 20 min can significantly increase the complexity index. A higher complexity index indicates higher heterogeneity of ultrasound images of plantar soft tissues. Homogeneous texture (low complexity index), however, suggests a sparse scatter distribution, which might be attributed to inflammation or fatty infiltration [27]. Only few studies have investigated the ultrasound images of pathological muscle tissues, and these studies showed low complexity of ultrasound images of these pathological muscle tissues [14]. Upper trapezius muscles containing active myofascial trigger points revealed significantly lower complexity than the control, suggesting that the local muscle fiber’s abnormal contractions and the formation of trigger points led to lower complexity [14]. Therefore, we postulated that the low complexity index might be indicative of an abnormal state of soft tissue. A session of 20 min walking at a speed of 3.6 mph increased the complexity index, which indicated such exercise intensity might be a protective factor against abnormal soft tissue status.

From the perspective of skin blood flow response to different walking intensities, Wu and his colleagues observed that faster walking speed (5.4 mph) significantly increased plantar skin blood flow [5]. Impaired skin blood flow response to mechanical stress has been viewed as a predictor of foot ulcers [28,29,30]. Another study carried out by Lung et al. examined the elastography of plantar soft tissues after walking at different speeds and durations and demonstrated that 3.6 mph walking for 10 and 20 min could significantly reduce plantar soft tissue stiffness, which is considered to be a protective factor. Thus, this study recommends people at risk for foot ulcerations walk at 3.6 mph rather than at 1.8 mph [31]. The results of this study indicated that slow walking speed might not be good enough for cardiovascular function or for foot ulcer prevention. Furthermore, the results regarding the spectral analysis of skin blood flow oscillations showed that metabolic, neurogenic, myogenic, respiratory, and cardiac mechanisms contributed to the plantar skin microvascular control in faster walking. On top of these previous studies researching the impact of various walking speeds and durations on plantar soft tissue, when considering microvascular function, plantar pressure, and soft tissue biomechanics, a moderate walking speed (3.6 mph) at a longer duration (20 min) might be more appropriate for people who are more likely to develop plantar skin injuries [5,32,33].

In medical image processing, the aim of pre-processing is to enhance characteristics for analysis. Usually, this is accomplished using the pixels in the space domain and the spectrums in the frequency domain, such as the approaches of image averaging, averaging filters, histogram equalization, image subtraction, threshold methods, and region growing [34]. To quantify image characteristics, methods were adopted to characterize statistical pixel-level, shape, texture, relational features, etc. The entropy concept is from thermodynamics and evaluates text features, including structural difference or complexity. The means of image characteristic recognition include minimum distance classification, the fuzzy classification method, and neural networks [35]. Because some spatial frequency components of the image are nonlinear, linear methods can ignore the hidden information in the image. Linear approaches usually measure quickly, but they can induce unambiguous outcomes if nonlinear relationships exist that lead to unsuitability for bio-signal applications. Because most biological systems are complex, nonlinear, and nonstationary, nonlinear analyses have been a suitable methodology in the past few decades [36]. Therefore, nonlinear image processing methods were proposed and applied to improve and quantify the nonlinear characteristics of the images [37,38,39,40,41,42,43]. One of these nonlinear methods for image complexity measurement, MSE_2D_, was proposed and applied in several fields. It was reported to be a useful nonlinear tool for image processing [21,22].

There are limitations to this study. First, the interpretation of the current findings requires caution due to the small sample size and healthy study population. We cannot conclude confidently enough to recommend a walking exercise intensity for people at risk of developing foot ulcers (e.g., diabetes). However, the value of this study is that when prescribing walking exercise to people with a risk of foot ulcers, clinicians may need to reconsider appropriate exercise intensity, not just moderate-to-vigorous intensity based on the guidelines, to achieve the purpose of enhancing cardiovascular function and preventing skin breakdown. A larger sample size of people at risk of foot ulcers needs to be studied in the future to increase the statistical power and clinical benefits. Second, there is a need to investigate the effects of a longer period (>20 min) of walking exercise because long walking times might cause different physiological responses. Third, the study by Valencia et al. [44] indicates that MSE has two limitations: artificial MSE reduction due to the coarse graining procedure, and the introduction of spurious MSE oscillations due to the suboptimal procedure for the elimination of the fast temporal scales. In our future study, we may use a high-resolution ultrasound probe to obtain high-resolution images in order to decrease the effects of artificial MSE reduction when using the coarse graining procedure.

## 5. Conclusions

MSE_2D_ was used to analyze plantar ultrasound images after different walking speeds and durations. Our study demonstrated that MSE_2D_ is useful for characterizing ultrasound image information. A walking speed of 3.6 mph for 20 min could be the critical threshold that affects image irregularity. Therefore, bidimensional multiscale entropy has good clinical practicality and guiding significance for formulating exercise prescription. Moreover, we propose that sessions of walking at 3.6 mph for 20 min might be a safe and effective exercise program to ameliorate cardiovascular health risks and simultaneously reduce the risk of foot ulcers.

## Data Availability

Data sharing not applicable.
